# Small Bowel Imaging in Managing Crohn's Disease Patients

**DOI:** 10.1155/2012/502198

**Published:** 2012-02-14

**Authors:** Jörg G. Albert

**Affiliations:** Department of Medicine I, Johann Wolfgang Goethe University Frankfurt, Theodor-Stern-Kai 7, 60590 Frankfurt, Germany

## Abstract

The small bowel is essential to sustain alimentation and small bowel Crohn's disease (CD) may severely limit its function. Small bowel imaging is a crucial element in diagnosing small bowel CD, and treatment control with imaging is increasingly used to optimize the patients outcome. Thereby, capsule endoscopy, Balloon-assisted enteroscopy, and Magnetic resonance imaging have become key players to manage CD patients. In this review, role of small bowel imaging is detailed discussed for use in diagnosing and managing Crohn's disease patients.

## 1. Introduction

Crohn's disease (CD) is a chronic inflammatory bowel disorder, and the patient might undergo recurring acute relapses. The disease is lifelong lasting and frequently manifests in the first decades of life. The small bowel is involved in more than two-thirds of patients. In some patients, refractory inflammation or chronic strictures of the small bowel are responsible for a debilitating course of the disease that might lead into malnutrition and a severely reduced quality of life. Therefore, the small bowel warrants special attention in diagnosing and treating CD. 

Ideally, diagnostic tools that reveal small bowel CD should be without objection to repeat them, easy and promptly to apply, and well tolerable. Many of these requirements are satisfied by modern diagnostics and imaging techniques. Recently, the small bowel has come within reach of easy-to-apply endoscopy, that is, capsule endoscopy (CE), balloon-assisted enteroscopy (BAE), and spiral enteroscopy. High-quality cross-sectional imaging complements endoscopy, and percutaneous ultrasound (US) and magnetic-resonance imaging (MRI) are at hand for an optimal steering instrument of treatment regimens.

Recently, endoscopy is increasingly used to control the effect of medical treatment in clinical practice of CD patients. When former studies were referring on symptom improvement, only [1], more and more studies include “objective” parameters such as endoscopic or radiologic findings to supervise the patient [2–5]. Primarily, the concept of endoscopic surveillance was established in the detection of postoperative recurrence some 20 years ago [3]. With the evidence of complete mucosal healing in modern immunomodulation therapies, arguments for endoscopic treatment control are getting stronger [4, 5].

This review updates on new small bowel imaging methods and their impact on managing small bowel CD.

## 2. Capsule Endoscopy

**Figure 1:**
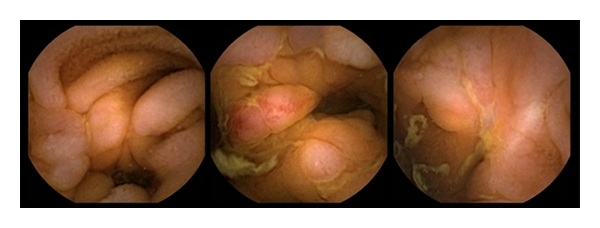
Crohn's disease of the small bowel in capsule endoscopy: multiple small ulcerations all over the ileum and jejunum, scarring alterations of the small bowel.

Small bowel video CE offers a noninvasive and easy-to-apply investigation of the small bowel. The video capsule is ingested and passes the intestinal tract by use of natural peristalsis. Thereby, images are continuously acquired until battery exhaustion and are registered at the storage device that the patient wears as his belt. Imaging data are afterwards reviewed by a specialist on a computer workstation. Most systems offer online visualization of the endoscopic procedure, but this feature is mainly used to confirm passage progress and not to detect a lesion. At present, there are several commercially available CE systems on the market that differ somehow in terms of technical details or software features: PillCam SB2, Given Imaging, Yoqneam, Israel (http://www.givenimaging.com/); EndoCapsule, Olympus Europe GmbH, Hamburg, Deutschland, (http://www.olympuseuropa.com/endoscopy/); CapsoVision, Saratoga, Ca, USA, (http://www.capsovision.com/); OMOM, Chongqing Jinshan Science, Beijing, China, (http://www.cqjs.net/); Miro-Cam, IntroMedic, Seoul, Korea (http://www.intromedic.com/). In the USA, only the PillCam SB2 and the EndoCapsule are currently approved by the US Food and Drug Administration, and in Europe, all the four systems can be purchased in most countries. Widely spread is the PillCam SB 2 capsule that has been used for almost all studies on CE in CD. The PillCam SB2 uses a CMOS chip with a resolution of 0.1 mm at a magnification of 1 : 8. Battery life is 8 h (SB 2) to about 12–16 hours (SB 2L); Table 1; Figure 1.

CE is usually not used in patients with intestinal strictures or potential stenosis for fear of retention. Dysphagia is a relative contraindication for CE, but the capsule might be placed in the duodenum by means of esophagogastroduodenoscopy (EGD) in case of swallowing disorders or gastroparesis. Pregnancy and any implanted medical device are still considered contraindications, but there is an increasing experience with the use of the PillCam capsule in patients with cardiac pacemakers or defibrillators. Thereby, interference for disadvantage of the patient has not been reported [22]. The main limitation of the capsule is the lack to take biopsies or to perform interventions, the difficulty to exactly localize identified lesions, and to control its movement. By administering patency capsule before doing video CE, capsule retention can be reliably prevented [23, 24]. The patency system (i.e., given AGILE patency capsule) is designed to determine which patients with known or suspected intestinal strictures can safely ingest a video capsule endoscope. The capsule is of similar size to the endoscopy capsule, but is made of lactose and barium and dissolves within 32 to 72 hours of entering the GI tract. Excretion of the intact capsule without symptoms predicts the uncomplicated passage of the wireless capsule endoscope. 

## 3. Balloon-Assisted Enteroscopy (BAE) 

Balloon-assisted enteroscopy (BAE) was first introduced in 2003 [25]. BAE allows deeper intubation of the SB compared with push enteroscopy (PE) and ileocolonoscopy (IC). BAE involves push-and-pull maneuvers for deep intubation of the small bowel [26], and single- and double-balloon enteroscopes (SBE and DBE) are presently available [27]; Table 2. 

Rate of complete small bowel investigations seems to be more regularly achievable using double-balloon instead of single-balloon technique as reported in randomized studies (Table 3), but therapeutic impact was similar to SBE and DBE. Preference for SBE or DBE depends on the experience and predilection of the endoscopic centre. 

Carbon dioxide insufflation instead of using ambient air increases intubation depth and significantly reduces inconvenience of the patient and may therefore be preferred for all balloon-enteroscopy interventions [28, 29]. Complication rates are low in diagnostic BAE (<5%) and include pancreatitis (<1%), bleeding, and perforation, and rate of complications increases in therapeutic interventions [30]. 

## 4. Spiral Enteroscopy (SE) and Others 

Enteroscopy with the Endo-Ease system (Spirus Medical, Stoughton, MA) uses a spiral-shaped overtube of 118 cm with a spiral of 0.55 cm high and 22 cm long and can be used with enteroscopes of less than 9.4 mm in diameter. The enteroscope is advanced or withdrawn with rotatory clockwise and counterclockwise movements of the spiral. Endoscopy of the small bowel by SE is reported to be safe [31] and seems to reduce the examination time, but the insertion depth is minor in comparison to DBE [32–34]. In Crohn's disease patients, SE has rarely been performed up to now. 

## 5. Radiology in Imaging Small Bowel Crohn's Disease 

Visualization of the small bowel with cross-sectional imaging methods requires distension of the intestines to identify the configuration of the bowel loops and to improve characterization of the bowel wall with luminal contrast. This is achieved by inserting a nasojejunal tube into the proximal small bowel (enteroclysis) or with oral intake of the luminal contrast medium (enterography). Conventional fluoroscopy (small bowel follow-through and small bowel enteroclysis) has thereby almost completely been replaced by cross-sectional imaging methods. Computed tomography (CT) and MRI are available as CT-enterography/CT-enteroclysis (CT-E) or MR-enterography/MR-enteroclysis (MR-E) with oral intake providing similar quality images but with an improved patient comfort over tube-assisted infusion of enteral contrast [15, 35, 36]. MR and CT equally provide excellent information on inflammatory alterations of the small bowel and also of extraluminal complications (abscess, fistula), thus adding useful information on endoscopic investigations; Table 4. 

Large lifetime doses of radiation are a concern particularly in young patients. CD patients are at risk for an increased exposure, and the often young age at the initial diagnosis has a significant influence on lifetime risk [37]. Radiation doses of more than 100 mSv may be observed in some patients. Lack of radiation exposure and excellent soft tissue contrast argue for use of MRI in CD patients and against fluoroscopy or CT [38–42]. 

## 6. Percutaneous Ultrasonography for Detection of Small Bowel Crohn's Disease 

Percutaneous ultrasonography (US) is useful to detect small bowel CD and to reveal extraintestinal complications, for example, abscess or fistula. Sensitivity of the technique is improved with the use of enteral contrast medium, such as polyethylene glycol [41]. Overall accuracy might be minor to endoscopy, but an experienced investigator can beneficially use US as an initial diagnostic tool for managing CD patients [42]. 

## 7. Diagnosing Small Bowel Crohn's Disease: Endoscopy in Comparison to Radiologic Imaging 

Small bowel endoscopy and MR-E/CT-E are accepted as a diagnostic standard to evaluate small bowel CD, but diagnostic sequence and clear definition of applying endoscopy versus cross-sectional imaging is under debate. Meta-analysis of studies comparing diagnostic yield and value of CT-E, MR-E, CE, and other methods were published in 2006 [43] and 2010 [44]. Thereby, higher sensitivity of endoscopic methods, for example, CE, to detect small bowel lesions was clearly demonstrated; Table 5. 

Next to high sensitivity, endoscopy has an excellent negative predictive value to exclude manifestation of small bowel CD. But endoscopic and radiologic findings are far from being pathognomonic, and small bowel ulceration may similarly be compatible with chronic inflammatory, neoplastic, and infectious origin or might be secondary to NSAID intake. In a cohort of patients who were suspected to be afflicted with small bowel CD, 37% of 102 patients were initially diagnosed with small bowel ulcerations in CE, but in only 13% the diagnosis of CD was maintained at one year of followup [45]. This reflects the “old” wisdom, that diagnosing CD is based on many clinical data including follow-up of the patient. Even if some features of small bowel lesions might rather suggest CD (irregular and longitudinal ulcerations, multiple locations, and cobble stone aspect of the small bowel) than NSAID use (circular ulcerations, webs) or neoplasia (circumscribed lesion), these identifiers must be interpreted cautiously before labeling a patient to be affected by CD. 

## 8. Diagnosing Small Bowel Crohn's Disease: Suspected Crohn's Disease

In suspected CD, ileocolonoscopy is still the reference standard in the diagnostic algorithm. Consensus conferences therefore advised to keep to a specific diagnostic sequence in suspected CD: first, ileocolonoscopy is used to diagnose ileitis terminalis or colitis, this is followed by cross-sectional imaging to identify proximal CD or extraenteric lesions, and CE is regarded a final identifier for detection of small bowel lesions that are reason for unexplained symptoms [46, 47]. These recommendations have not yet considered recent study results demonstrating equal validity to detect terminal ileitis for CT-E, MR-E, and CE. Moreover, significantly better detection of proximal small bowel involvement may be expected for CE [15]. Moreover, high negative predictive value of CE of 90 to 100% suggests using CE to exclude CD in suspected disease cases. Therefore, CE could be considered an early step in suspected CD and nonconclusive colonoscopy in the future. To exclude neoplastic and infectious disease, flexible enteroscopy should regularly be performed to take biopsies in lesions found by CE. Cross-sectional imaging (e.g., US, MRI) is indicated to screen for extraintestinal disease. Studies are not yet available that might support the use of CE to investigate the small bowel plus the colon in a “one-step shopping” approach using the colon capsule endoscope. 

## 9. Diagnosing Small Bowel Crohn's Disease: Established Crohn's Disease

In established CD, value of cross-sectional imaging (e.g., MRI) surpasses endoscopic information in many clinical scenarios such as the septic patient, and acute onset of severe complaints and pain. Severe inflammations of bowel segments, suppurative disease, and conglomerate tumor or fistulae are thereby detected. Performance of ileocolonoscopy and/or small bowel endoscopy is necessary to discriminate inflammatory from chronic-scarring bowel changes or strictures. Indication for BAE has to be balanced against CE preferring flexible endoscopy in suspicion of high grade strictures or in case the “patency capsule” failed to pass the intestines. 

## 10. Colitis with an Unclassified Type of Inflammatory Bowel Disease (IBDU) 

In colitis with an unclassified type of inflammatory bowel disease (IBDU), small bowel inflammation could be the clue to confirm the diagnosis of CD in some patients. The diagnosis of IBDU had been revised and changed to CD in 15% of 120 patients in one study in which small bowel ulcerations were detected [48, 49]. Discrete findings should not mislead to revise the diagnosis, despite [50], and negative small bowel CE might not completely exclude CD—for example, of the colon [49]. 

In the postoperative situation, endoscopic surveillance of the patient has been recommended [47], and endoscopy seems essential to discriminate inflammatory from noninflammatory bowel alterations. CE might replace ileocolonoscopy to detect recurrence: accuracy of CE to detect inflammation near to the anastomosis is similar to conventional colonoscopy, but proximal disease is exclusively visualized by CE [41, 51, 52]. 

## 11. Endoscopic Treatment in Crohn's Disease 

Flexible endoscopy offers treatment options for CD strictures in selected cases. Balloon dilation has been expanded from colonic and anastomotic stricture to the whole of the small bowel by use of BAE, and symptomatic small bowel strictures may be treated at anastomotic and nonanastomotic sites with a justifiable risk profile (Table 6). Balloon dilation might well be repeated in recurring strictures. 

## 12. Imaging for Treatment Control 

Imaging is used to control treatment and to assess prognosis in Crohn's disease patients, but this is far from routine practice. New concepts of modern treatment regimes that aim to attain complete mucosal healing support to verify treatment success using diagnostic procedures and a new verve for imaging to help managing CD patients may recently be noticed [53]. Nevertheless, studies keep relying on clinical outcome without supporting subjective endpoints such as imaging data [1]. But introduction of objective study outcomes, for example, by assessing endoscopic activity of CD before and after treatment is increasingly reported, for example, in the postoperative situation [2–5]. Interestingly, we know for over 20 years that endoscopic activity predicts course of the disease in high-risk, postoperative patients [3], and endoscopic treatment control in this patient group is well established [54]. Control of mucosal healing has been used to assess treatment efficacy in ulcerative colitis, but in CD, this concept has only emerged after biological therapies have been evaluated in clinical trials. Even if mucosal healing has been shown to reduce hospitalizations and surgery, strong correlation of mucosal healing and symptom improvement has not been proven yet [5]. Today, in clinical practice, endoscopy is used to assess mucosal healing in patients with persistent symptoms despite therapy and when treatment discontinuation is considered. Further studies have to provide the value of managing all CD patients with endoscopic or radiologic imaging. 

## 13. Conclusion 

Small bowel imaging is a crucial element in diagnosing small bowel CD, and CE, BAE, and MRI have become key players to manage CD patients. Treatment control is strongly advised in the patient who had formerly undergone bowel resection, but is increasingly used to testify treatment success in many patients. Endoscopy is indispensable for diagnosis at first presentation, and cross-sectional imaging is the first-line diagnostic means in established disease and presentation with severe disease. Thus, complementary use of cross-sectional imaging and endoscopy is essential for the best benefit of the patient. 

## Figures and Tables

**Table 1:** Four capsule endoscopes are available at present.

Capsule	Company	Size (mm)	Frame rate (Images/s)	Field of view	Acquisition time (hours)
PillCam SB 2	Given Imaging, Israel	26 × 11	2	156°	8 (SB 2); ca. 12–16 (SB 2L)
EndoCapsule	Olympus, Japan	26 × 11	2	145°	> 8
MiroCam	IntroMedic, Korea	25 × 11	3	—	> 11
OMOM	Chongqing Jinshan Science, China	28 × 13	2 or 1	140°	8
CapsoVision	CapsoVision Inc.	31 x 11.3	0–5	360°	15

**Table 2:** Balloon-assisted enteroscopy. Technical data of the scopes that are presently available.

Device	Company	Working channel (mm)	Length (mm)	Working length (mm)	Diameter of distal part (mm)	Length of overtube (mm)	Diameter of overtube (mm)
DBE EN-450P5	Fuji	2.2	2300	2000	8.5	1450	12.2 (outer diameter) 8.7–10 (inner diameter)
DBE EN-450T5	Fuji	2.8	2300	2000	9.4	1450	13,2 (outer diameter) 9.8–10.8 (inner diameter)
SBE SIF-Q180	Olympus	2.8	2300	2000	9.2	1320	13 mm (ST-SB1)

**Table 3:** Comparison of single-balloon (SBE) or double-balloon technique (DBE) in prospective, randomized studies.

Author	Year	*n*	Complete enteroscopy		Diagnostic yield		Therapeutic yield	
			SBE	DBE	SBE	DBE	SBE	DBE
May et al. [6]	2010	100	22%	66%	42%	52%	42%	52%
Domagk et al. [7]	2011	150	11%	18%	37%	43%	5%	9%
Takano et al. [8]	2011	38	0%	57%	61%	50%	28%	35%

**Table 4:** Comparing pros and cons of CT versus MRI in use in Crohn's disease patients; CT-enterography/CT-enteroclysis (CT-E) or MR-enterography/MR-enteroclysis (MR-E).

CT-E		MR-E	
Pro	Con	Pro	Con
High resolution in contrast-rich objects (e.g., bone versus parenchyma)		Excellent contrast in soft tissues; contrast uptake into tissues is well visualized	
Fast acquisition time, minor motion artifacts			Increased acquisition time, prone to motion artifacts
Widely spread technique			Higher cost and less available
Abdomen and pelvis are a one-step investigation			Abdomen/pelvis are usually different examination protocols
	Radiation exposure	No radiation exposure	
	Specific side effects of contrast medium		Specific side effects of contrast medium
			Restriction in pace-maker patients and so forth

**Table 5:** Comparison of diagnostic yield or sensitivity in cross-sectional imaging and endoscopy in diagnosing small bowel Crohn's disease; CTE: computed tomography enterography; MRI: magnetic resonance imaging.

Author	Year of publication	*N*	Capsule endoscopy	Comparator (cross-sectional imaging)		Statistical significance
Eliakim et al. [9]	2004	35	77%	CTE	20%	*P* < 0.05
Voderholzer et al. [10]	2005	41	25/41 (61%)	CTE	12/41 (29%)	*P* = 0.004
Hara et al. [11]	2006	17	12/17 (71%)	CT	9/17 (53%)	n.s.
Solem et al. [12]	2008	28	83%	CTE	83%	n.s.
Albert et al. [13]	2005	52	25/27 (93%)	MRI	21/27 (78%)	n.s.
Golder et al. [14]	2006	18	12/18 (66%)	MRI	1/18 (5%)	*P* = 0.016
Jensen et al. [15]	2011	93	100%	MRI and CTE	81% (MRI) 76% (CTE)	*P* < 0.05 (for proximal small bowel)

**Table 6:** Balloon dilation for symptomatic small bowel strictures with the use of balloon-assisted enteroscopy; Small bowel strictures in anastomotic and nonanastomotic Crohn's disease.

Author	Year	*n*	Balloon dilation			Complication
			Technical success	Clinical response	Failed	
Yamamoto et al. [16]	2004	6	6	6	6	None
Pohl et al. [17]	2007	19	8	6	13	None
Fukumoto et al. [18]	2007	23	22	17	2 (Surgery) 4 (repeated dilation)	None
Despott et al. [19]	2009	11	9	8	2	Perforation (*n* = 1)
Hirai et al. [20]	2010	25	18	18	7	*n* = 2
Kondo et al. [21]	2010	12	8	7	1	None

## Abbreviation

APC: Argon plasma coagulation
BAE: Balloon-assisted enteroscopy
CE: Capsule endoscopy
CT: Computed tomography
CT-E: CT-enteroclysis/enterography
DBE: Double-balloon enteroscopy
EGD: Esophagogastroduodenoscopy
ERC: Endoscopic retrograde cholangiography
IBDU: Unclassified type of inflammatory bowel disease
MR-E: MR-enteroclysis/enterography
MRI: Magnetic resonance imaging
NSAID: Nonsteroidal anti-inflammatory drugs
SBE: Single-balloon enteroscopy
TTS: Through the scope
US: Percutaneous ultrasonography.
